# Short-term inhibition of glutamine synthetase leads to reprogramming of amino acid and lipid metabolism in roots and leaves of tea plant (*Camellia sinensis* L.)

**DOI:** 10.1186/s12870-019-2027-0

**Published:** 2019-10-15

**Authors:** Mei-Ya Liu, Dandan Tang, Yuanzhi Shi, Lifeng Ma, Yan Li, Qunfeng Zhang, Jianyun Ruan

**Affiliations:** 1 Key Laboratory of Tea Plant Biology and Resources Utilization (Ministry of Agriculture), Tea Research Institute, Chinese Academy of Agricultural Sciences, Hangzhou, 310008 China; 2 Max Planck Institute of Molecular Plant Physiology, 14476 Potsdam-Golm, Germany

**Keywords:** Glutamine synthetase, Methionine sulfoximine, Quality-related compounds, Amino acids, Lipids, Tea plant

## Abstract

**Background:**

Nitrogen (N) nutrition significantly affected metabolism and accumulation of quality-related compounds in tea plant (*Camellia sinensis* L.). Little is known about the physiological and molecular mechanisms underlying the effects of short-term repression of N metabolism on tea roots and leaves for a short time.

**Results:**

In this study, we subjected tea plants to a specific inhibitor of glutamine synthetase (GS), methionine sulfoximine (MSX), for a short time (30 min) and investigated the effect of the inhibition of N metabolism on the transcriptome and metabolome of quality-related compounds. Our results showed that GS activities in tea roots and leaves were significantly inhibited upon MSX treatment, and both tissue types showed a sensitive metabolic response to GS inhibition. In tea leaves, the hydrolysis of theanine decreased with the increase in theanine and free ammonium content. The biosynthesis of all other amino acids was repressed, and the content of N-containing lipids declined, suggesting that short-term inhibition of GS reduces the level of N reutilization in tea leaves. Metabolites related to glycolysis and the tricarboxylic acid (TCA) cycle accumulated after GS repression, whereas the content of amino acids such as glycine, serine, isoleucine, threonine, leucine, and valine declined in the MXS treated group. We speculate that the biosynthesis of amino acids is affected by glycolysis and the TCA cycle in a feedback loop.

**Conclusions:**

Overall, our data suggest that GS repression in tea plant leads to the reprogramming of amino acid and lipid metabolic pathways.

## Background

Glutamine synthetase (GS, EC 6.3.1.2), a key enzyme in the GS-glutamine-2-oxoglutarate amidotransferase (GS-GOGAT) pathway, assimilates ammonium either immediately following its uptake by roots or after nitrate has been reduced to nitrite in either roots or leaves [1]. In addition to the fundamental roles of the GS-GOGAT pathway in N assimilation, products of the GS-GOGAT pathway act as signals or precursors for further regulation of primary and secondary metabolism in plants. Glutamate, which is incorporated into other amino acids through the action of aminotransferases or transaminases, is the net outcome of the GS-GOGAT cycle [2, 3]. Specific amino acids serve as precursors for all nitrogen (N)-containing organic molecules, such as proteins, chlorophyll, cytochrome/phytochrome, secondary metabolites and nucleic acids [3]. In tea plant, the total amount of free amino acids in leaves accounts for 1–5% of the dry weight, and each amino acid provides a unique flavor and/or aroma to brewed tea [4, 5]. Furthermore, a large number of N-containing amides, pigments, and secondary metabolites determine the quality of tea [4]. GS is, therefore, essential for primary and secondary metabolism as well as for the formation of tea quality [6, 7].

In addition to the role of GS in N assimilation, GS also affects downstream metabolism. In rice (*Oryza sativa* L.), *OsGS1;1* mutant and wild-type rice show quantitative differences in the metabolic profiles of sugars, amino acids, tricarboxylic acid (TCA) cycle products, and secondary metabolites [8]. This suggests that GS plays an important role in coordinating the global metabolic network in plants, when ammonium or nitrate is supplied as the N source.

Lipids are major subcellular components and are sensitive to environmental cues, including N status [9]. For decades, researchers have shown that N status affects the remodeling of lipids [10, 11]. In *Arabidopsis thaliana*, N limitation leads to a coordinated breakdown of galactolipids and chlorophyll, with the deposition of specific fatty acid phytyl esters in thylakoids and plastoglobules of chloroplasts [10]. Recently, Liu et al. [11] reported that N availability affects lipid metabolism in mature leaves and new shoots of tea plants. Since lipids determine the flavor and aroma of brewed tea [12], investigation of the relationship between GS activity and lipid metabolism in tea plants is of great interest.

Tea plant is one of the most important cash crops in tropical and subtropical countries [13]. The use of N fertilizer is critical for the yield and quality of tea. Quality-related metabolites (e.g. amino acids, catechins, caffeine, lipids...) in tea leaves are significantly affected by the level and form of N fertilizer used. Concentrations of free amino acids, particularly theanine which is mainly synthesized in roots and then transported to leaves, are considerably higher in ammonium-fed tea plants than in nitrate-fed plants [14]. Most of the catechins and caffeine are generated in tea plant leaves, and their contents are different in these 2 N forms [14, 15]. Under long-term use, the level of N application also affects the biosynthesis of lipids, which are thought to be responsible for the generation of flavor and aroma compounds [11]. Studies on transcriptome analysis of tea plant have identified several N utilization genes in response to different levels and forms of N fertilizer; *AMT*, *NRT*, and *AQP* genes are key for N uptake, while *GS* and *GOGAT* genes are important for N assimilation [16, 17]. Previous studies concerning N treatment in tea plant usually sustained for a long period [14, 16, 18]. Limitation of N supply for a long time could cause severe damage to tea plant with inhibition of plant growth and chlorosis in tea leaves [18]. Consequently, these studies have not unambiguously explained the regulation process of metabolite biosynthesis in normally growing tea plants when N assimilation was restricted. Otherwise, effects of N application on plants can be recognized within minutes [19]. It was reported that the biosynthesis pathways of three main quality-related components (theanine, catechins and caffeine) were all regulated by nitrate and ammonium signaling within 30 min, which was different from the long time cultured treatments of these 2 N forms in tea plant [15]. GS is a key enzyme that regulates N assimilation in plants; inhibition of GS by methionine sulfoximine (MSX) for 30 min not only affects the transcription levels of metabolic genes but also affects the metabolite content [20, 21]. Hence, in comparison with long-term N treatment, short-term MSX treatment of plants restricts N assimilation without causing N deficiency. Understanding the effect of short-term N treatment on tea plant will help unravel the metabolism of quality-related metabolites and provide further insight into the development of tea quality.

In this study, we investigated the effects of MSX, a specific GS inhibitor, on the metabolism of quality-related compounds, namely amino acids and lipids, in tea plants. To determine the effect of GS inhibition on the reprogramming of the transcriptome and metabolome, we performed metabolic profiling and RNA-seq analysis of roots and leaves of tea plants treated with or without MSX. Our study provides further insights into the mechanism of N assimilation and its regulation during the process of tea quality formation.

## Results

### MSX reduces GS activity in tea roots and leaves

**Fig. 1:**
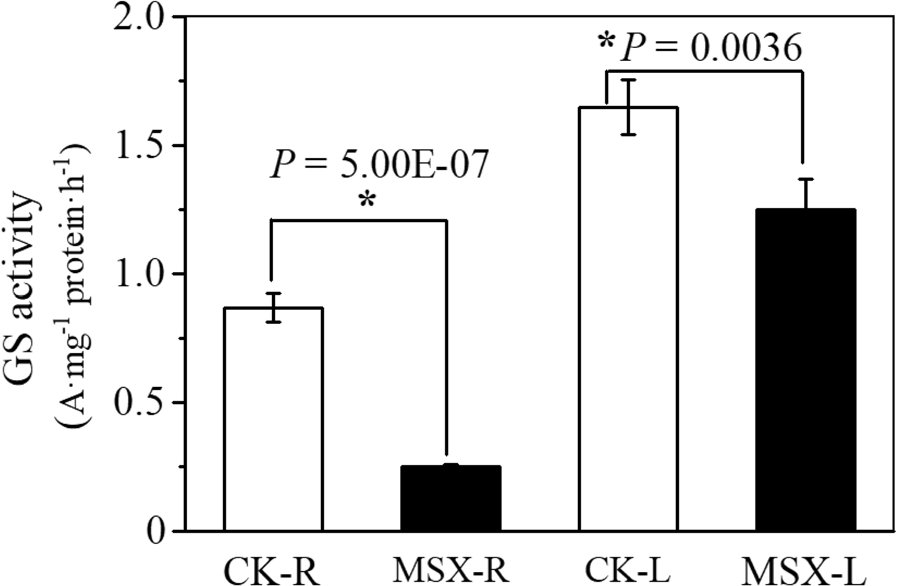
Effects of methionine sulfoximine (MSX) on the inhibition of glutamine synthetase (GS) in tea plant. The GS activity was expressed as the absorbance of mg^−1^ protein·h^−1^. CK-R/CK-L, roots (R) or leaves (L) of tea plants cultured with nutrient solution without MSX treatment. MSX-R/MSX-L, roots (R) or leaves (L) of tea plants subjected to MSX treatment. The asterisk indicates significant difference between MSX and CK groups of the same tissue (*P < 0.05). For each treatment 3 biological replicates were applied

MSX is a specific GS inhibitor, which has been proved to be able to block the assimilation of ammonia into amino acids in higher plants within 30 min [20, 22, 23]. To test the effectiveness of MSX in inhibiting GS activity, tea plants were treated with or without MSX for 30 min. In MSX-treated tea plants, GS activity was reduced by 71.1% in roots and by 24% in leaves in comparison with control plants (Fig. 1). This result suggests that MSX is effective to inhibit GS activity in tea plants.

### The overall transcriptome response to MSX in tea roots and leaves

**Fig. 2:**
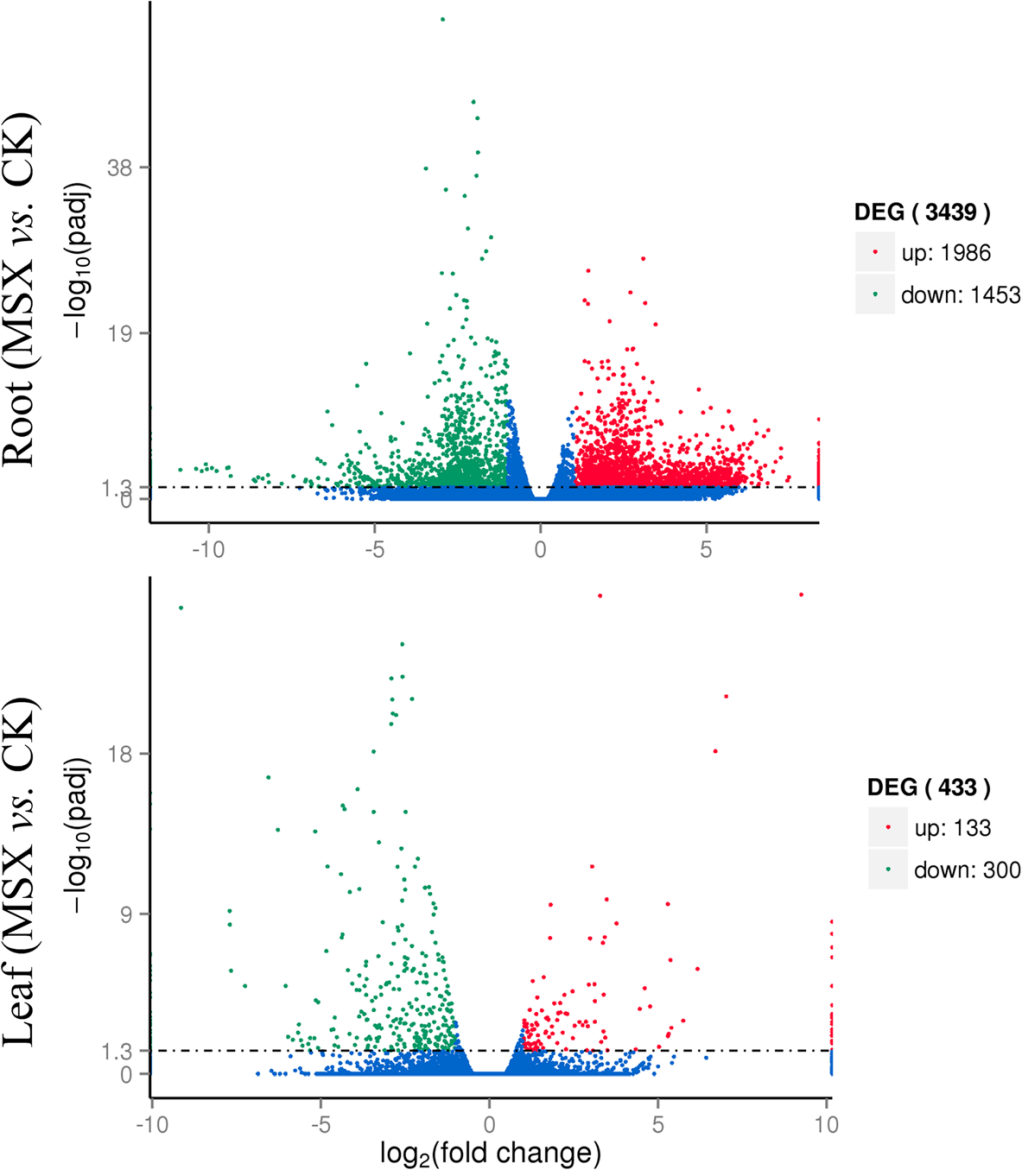
Volcano Plot depicting the number of differentially expressed genes (DEGs) between methionine sulfoximine (MSX) treated plants and control plants (CK). The red dots indicate up regulated genes in MSX vs. CK group, while the green dots indicate genes that are down regulated in MSX vs. CK group. The y-axis suggests the significant differences of each gene (padj < 0.05), whereas the x-axis indicates the fold change of each gene in MSX vs. CK group

**Fig. 3:**
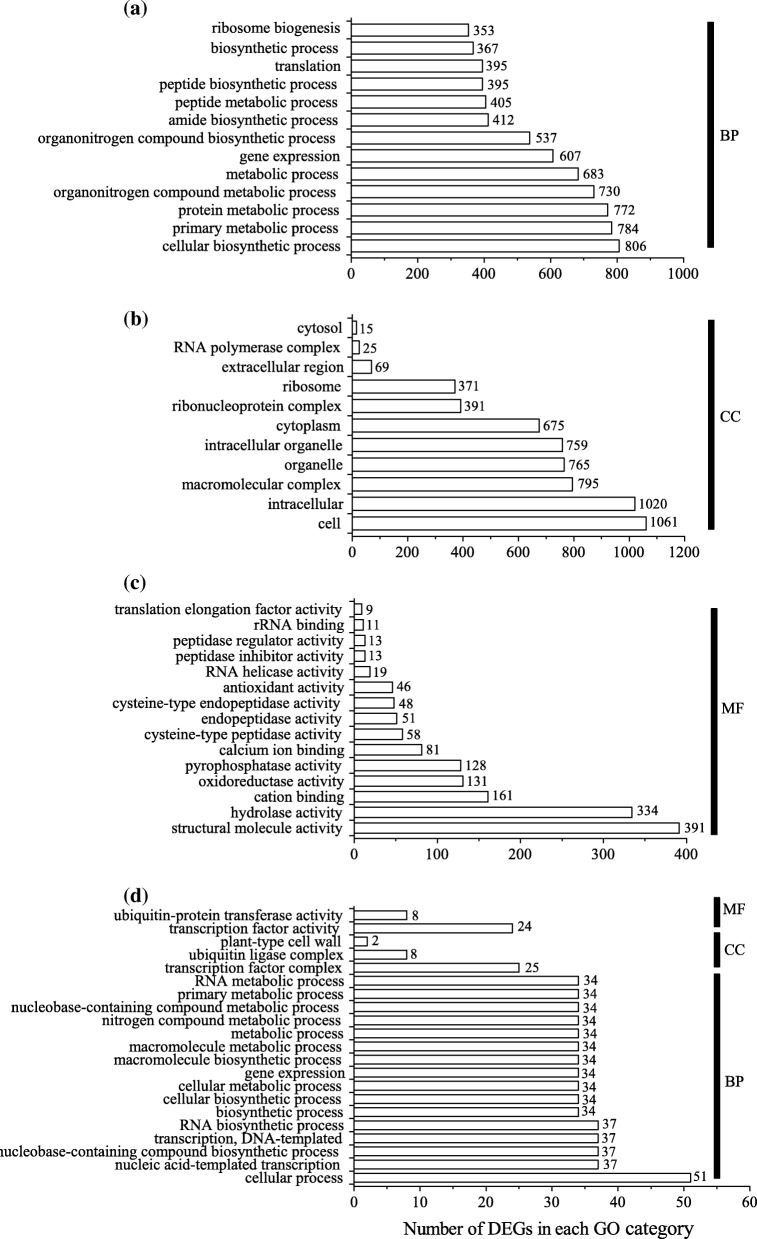
Enriched GO terms of the differentially expressed genes (DEGs) in roots (**a**, **b**, **c**) and leaves (**d**) between the methionine sulfoximine (MSX) treated plants and control plants. Genes were categorized based on GO annotation, and the number of DEGs (Arabic number beside each bar) in each category is displayed based on biological process (BP), cellular components (CC), or molecular function (MF)

Illumina sequencing with all the biological replicates of samples generated clean reads ranging from 45 × 10^6^ to 63 × 10^6^ (Additional file 1: Table S1). De novo assembly of unigenes from clean reads using Trinity software generated 326,169 unigenes (Additional file 2: Table S2). In roots, 3439 unigenes (the percentage of differentially expressed unigenes is 1.05%) were differentially expressed between the control and the MSX treated groups, among which 1986 unigenes were up-regulated while 1453 were down-regulated (Fig. 2, Additional file 3: Table S3). In leaves, only 433 genes (the percentage of differentially expressed unigenes is 0.45%) were differentially expressed with 133 genes up-regulated and 300 down regulated (Fig. 2, Additional file 4: Table S4). To gain insight into the functional categories of these DEGs, GO categories were assigned to all DEGs in roots and leaves (Fig. 3, Additional file 5: Table S5 and Additional file 6: Table S6). In roots, cellular biosynthetic process (806 DEGs in MSX vs. CK), primary metabolic process (784), protein metabolic process (772) and organonitrogen compound metabolic process (730) were the dominant categories in the biological process (Fig. 3a). Cell (1061) and intracellular (1020) were the major terms in the cellular components (Fig. 3b). According to the GO terms of the molecular function, the majority of DEGs appeared to be related to structural molecule activity (391) and hydrolase activity (334) (Fig. 3c). In tea leaves, there were 2 terms and 3 terms annotated for the molecular function and cellular components, respectively (Fig. 3d). And the most top category in biological process was cellular process (51 DEGs were annotated) (Fig. 3d). Furthermore, KEGG enrichment of the DEGs was also applied to dissect the major metabolic pathways affected by direct inhibition of GS (Additional file 7: Table S7, Additional file 8: Table S8). Although the 433 DEGs (Additional file 4: Table S4) identified in leaves could not be assigned to specific KEGG pathways, the 3439 DEGs in roots (Additional file 3: Table S3) were assigned to 64 metabolic pathways (Additional file 9: Table S9). The most altered pathways were clustered into KEGG classes of genetic information processing, carbohydrate metabolism, amino acid metabolism and lipid metabolism (Additional file 9: Table S9).

### MSX treatment affects amino acid biosynthesis in tea roots and leaves

**Fig. 4:**
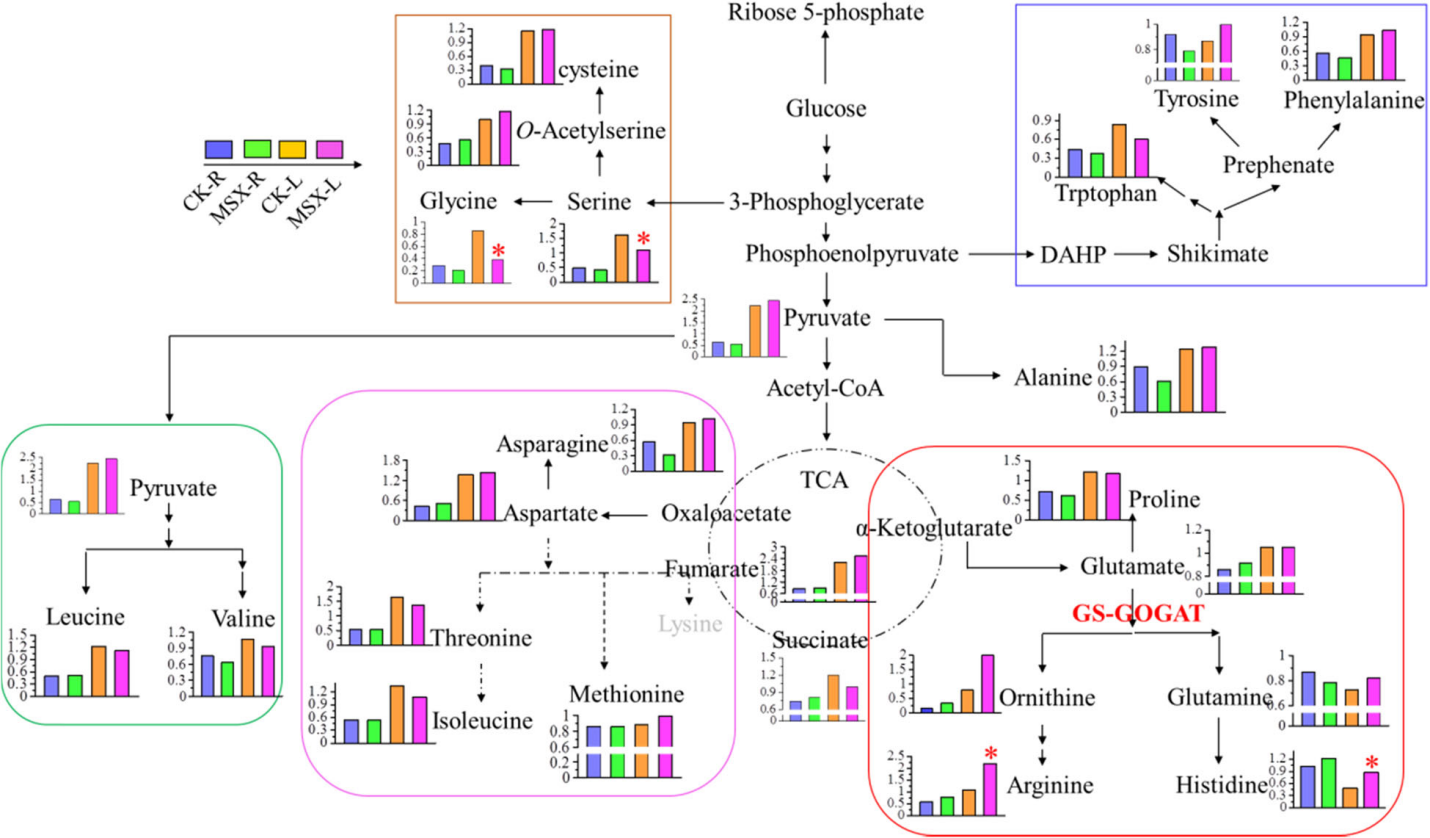
The reprogramming of amino acid metabolism affected by methionine sulfoximine (MSX) treatment. The length of each column indicates the relative intensity which represents the content of each detected metabolite. Metabolites in grey font were not detected in this study. The schematic flowchart showing the biosynthesis of 20 proteinogenic amino acids was drafted according to Coruzzi et al. (2015). Red asterisks indicate statistically significant differences between MSX and CK groups (*P < 0.05 in student’s *t*-test)

Free amino acids are flavor and aroma origin compounds in tea plant [12]. To uncover the overall metabolic changes in amino acids due to the direct inhibition of GS during N assimilation, primary metabolic profiling was performed via gas chromatography-mass spectrometry (GC-MS). A total of 155 primary metabolites were identified from the MSX and CK group (Additional file 10: Table S10), and significantly changed primary metabolites were listed in Table 1 (the percentage of significantly changed metabolites are 4.52% for root and 12.26% for leaf). The biosynthesis pathway of amino acids [24] and changes in 20 proteinogenic amino acids following MSX treatment are shown in Fig. 4. Contents of glutamine, histidine, ornithine, and arginine, which are synthesized directly downstream of the GS-GOGAT cycle, were higher in MSX-treated leaves than in control leaves (Fig. 4, red frame). Among the five amino acids in the aspartate family, contents of threonine and isoleucine were lower in MSX-treated leaves than in CK leaves and showed no difference in MSX-treated vs. control roots. Contents of asparagine, aspartate and methionine did not change after MSX treatment (Fig. 4, purple frame); similar results were obtained for leucine and valine (Fig. 4, green frame). Levels of both tyrosine and phenylalanine decreased in roots but slightly increased in leaves after the inhibition of GS with MSX (Fig. 4, blue frame). Alanine content was lower in MSX-treated roots but was unchanged in leaves following MSX treatment; the content of pyruvate, the substrate for alanine biosynthesis, also showed the same tendency (Fig. 4). Contents of glycine and serine were reduced in both roots and leaves of MSX-treated tea plants compared with the control group (Fig. 4, yellow frame).

**Table 1:** Significantly changed metabolites in tea roots and leaves treated with MSX. U.d. indicated undetected metabolites. FDRs (q-value) were calculated in a hierarchical way according to the metabolites groups

Compounds	Root			Leaf		
	Fold (MSX/CK)	P (t.test)	q-value	Fold (MSX/CK)	P (t.test)	q-value
Carbohydrate						
Fructose	0.91	0.13	0.33	0.97	0.21	0.35
Fructose-6-phosphate	0.88	0.50	0.56	1.11	0.23	0.33
Fucose	1.02	0.50	0.50	1.08	0.02	0.05
Glucose	0.99	0.04	0.20	u.d	u.d	u.d
Glucose-6-phosphate	0.76	0.22	0.37	1.33	0.01	0.03
Ribose	1.43	0.0002	0.002	1.41	0.0015	0.03
Sucrose	0.97	0.35	0.50	1.02	0.92	1.25
Maltose	1.02	0.41	0.51	1.18	0.04	0.14
Glyceric acid	1.09	0.16	0.32	1.00	0.01	0.05
Glycerol	1.35	0.08	0.27	1.02	0.61	0.76
Amino acid						
Methionine	1.00	0.42	0.48	1.12	0.25	0.30
O-acetyl-serine	1.18	0.12	0.35	1.16	0.03	0.08
Ornithine	1.59	0.03	0.35	2.00	0.01	0.06
Phenylalanine	0.82	0.06	0.35	1.10	0.06	0.12
Proline	0.86	0.40	0.51	0.97	0.57	0.57
Pyruvic acid	0.86	0.81	0.81	1.10	0.04	0.08
Serine	0.88	0.36	0.49	0.68	0.01	0.05
Threonine	1.01	0.50	0.55	0.83	0.30	0.35
Tryptophan	0.87	0.35	0.50	0.72	0.06	0.11
Tyrosine	0.86	0.16	0.41	1.15	0.02	0.08
Valine	0.84	0.34	0.52	0.87	0.10	0.15
Alanine	0.68	0.07	0.32	1.03	0.16	0.22
Asparagine	0.56	0.40	0.48	1.08	0.10	0.14
Glutamic acid	1.06	0.26	0.50	1.00	0.45	0.49
Glutamine	0.90	0.28	0.50	1.12	0.02	0.07
Glycine	0.80	0.25	0.52	0.58	0.0031	0.02
Histidine	1.19	0.08	0.31	1.82	0.0007	0.01
Isoleucine	0.97	0.70	0.73	0.81	0.04	0.09
Leucine	1.05	0.18	0.41	0.92	0.19	0.24
Aspartic acid	1.20	0.10	0.33	1.05	0.49	0.51
Citrulline	1.59	0.30	0.49	1.18	0.02	0.06
Arginine	1.39	0.0011	0.03	2.04	0.0001	0.0023
Cysteine	0.83	0.04	0.31	1.03	0.07	0.12
Organic acid						
Shikimic acid	0.93	0.12	0.36	0.98	0.28	0.28
Sinapic acid	0.90	0.97	0.97	1.32	0.14	0.19
Succinic acid	1.08	0.23	0.46	0.83	0.03	0.07
4-hydroxy-benzoic acid	1.08	0.47	0.71	1.08	0.06	0.12
4-Hydroxycinnamic acid	0.91	0.05	0.30	1.19	0.21	0.25
Adenine	1.14	0.04	0.48	1.45	0.02	0.06
Ascorbic acid	1.08	0.59	0.79	0.36	0.0001	0.0012
Benzoic acid	0.89	0.73	0.80	0.83	0.27	0.29
Fumaric acid	1.04	0.66	0.79	1.15	0.06	0.10
Hydroxyproline	0.74	0.11	0.44	1.28	0.01	0.06
Palmitic acid	0.92	0.25	0.43	1.08	0.13	0.20
Maleic acid	1.25	0.13	0.31	1.32	0.01	0.04

To confirm changes in amino acids obtained from the high throughput method, targeted quantitative analysis of some amino acids were conducted. Contents of all 13 free amino acids detected in leaves, except tyrosine and theanine, were significantly lower in MSX-treated leaves than in control leaves (Table 2). Compared with control leaves, contents of valine, aspartic acid and lysine were reduced in MSX-treated leaves by ca. 27%, and those of serine, alanine, cysteine and threonine were reduced by 32–36%. The most significantly decreased amino acids in MSX-treated leaves were leucine and phenylalanine, which were reduced by 45 and 64%, respectively (Table 2). Glutamate and theanine were the most abundant free amino acids detected. Interestingly, the content of theanine in MSX-treated plants was higher than that in control plants, whereas the content of glutamate was slightly decreased following MSX treatment (Table 2). Changes of serine, threonine, tyrosine, valine, alanine, histidine, leucine and arginine showed the same tendency as detected by GC-MS.

**Table 2:** Contents of free amino acids (mg/g fresh weight) in the leaves of tea plant treated with methionine sulfoximine (MSX). The value shown is mean ± standard deviation (SD). CK indicates plants grown with nutrient solution without MSX treated, while MSX indicate plants treated with MSX. The asterisk indicates significant difference between MSX and CK group (*P < 0.05, **P < 0.01 in student’s *t*-test). For each treatment 3 biological replicates were used

	CK	MSX	P (*t*-test)
Theanine	2.98 ± 0.06	3.92 ± 0.23	0.18
Glutamate	0.47 ± 0.04	0.40 ± 0.02	0.11
Valine	0.22 ± 0.01	0.16 ± 0.02*	0.02
Serine	0.21 ± 0.03	0.13 ± 0.01*	0.02
Aspartic acid	0.18 ± 0.01	0.13 ± 0.01*	0.03
Alanine	0.17 ± 0.01	0.11 ± 0.01**	0.0016
Arginine	0.16 ± 0.05	0.20 ± 0.01	0.83
Cysteine	0.12 ± 0.01	0.08 ± 0.01**	0.0047
Methionine	0.12 ± 0.01	0.10 ± 0.01	0.06
Glycine	0.12 ± 0.01	0.12 ± 0.02	0.48
Threonine	0.11 ± 0.01	0.08 ± 0.01*	0.03
Leucine	0.11 ± 0.01	0.06 ± 0.01*	0.02
Histidine	0.11 ± 0.01	0.13 ± 0.01	0.82
Tyrosine	0.07 ± 0.01	0.05 ± 0.01	0.06
Lysine	0.03 ± 0.01	0.02 ± 0.01*	0.03
Phenylalanine	0.02 ± 0.01	0.01 ± 0.01**	0.0003

### MSX reduces theanine hydrolysis in tea leaves

**Fig. 5:**
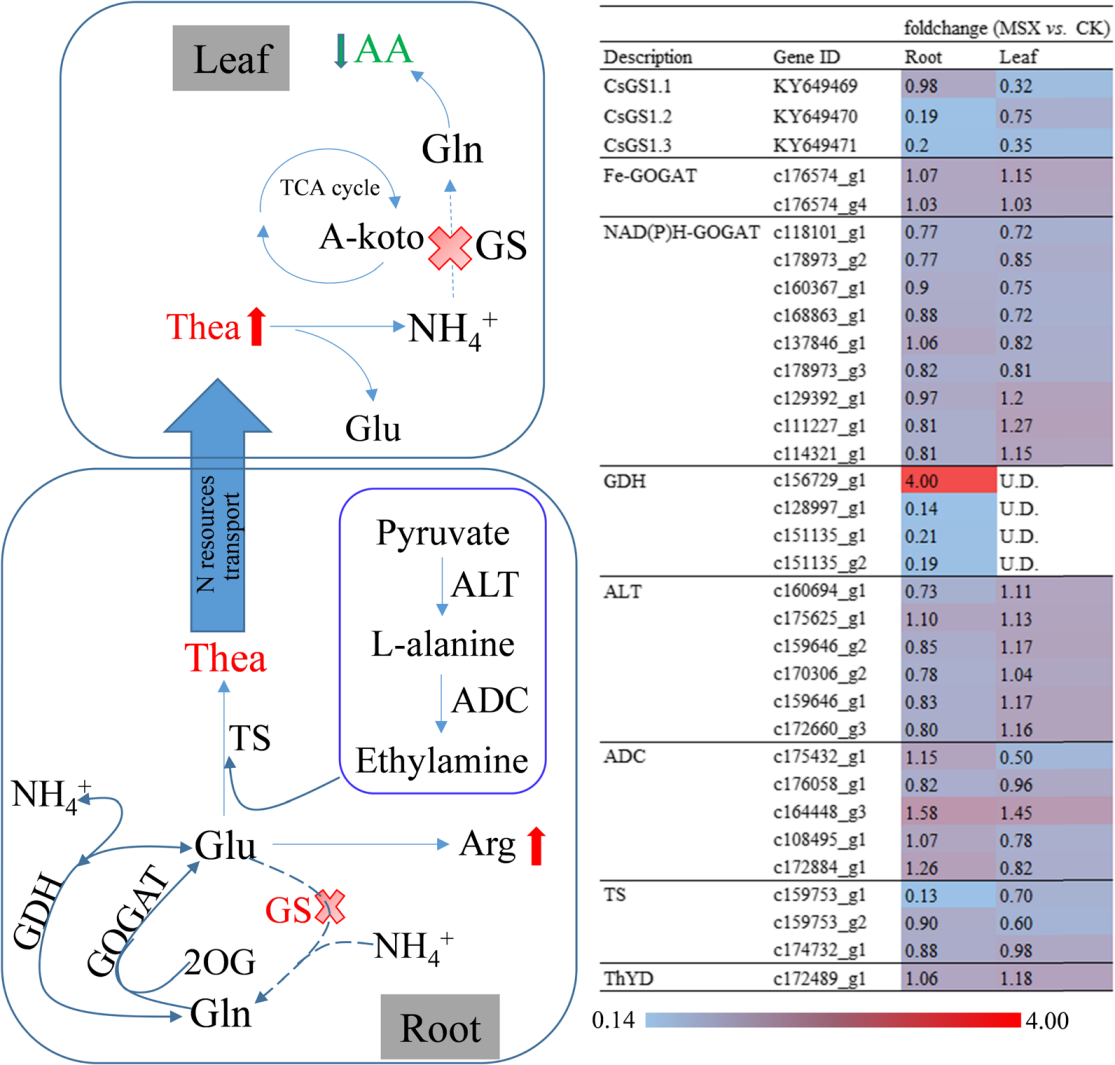
Effect of methionine sulfoximine (MSX) treatment on theanine metabolism in roots and leaves of tea plants. Thea, theanine; Glu, glutamic acid; Gln, glutamine; Arg, arginine; GS, glutamine synthetase; Fe-GOGAT, ferredoxin-dependent glutamate synthase; NAD(P)H-GOGAT, NAD(P)H-dependent glutamate synthase; GDH, glutamate dehydrogenase; ALT, alanine aminotransferase; ADC, arginine decarboxylase, TS, theanine synthetase; ThYD, theanine hydrolase

**Fig. 6:**
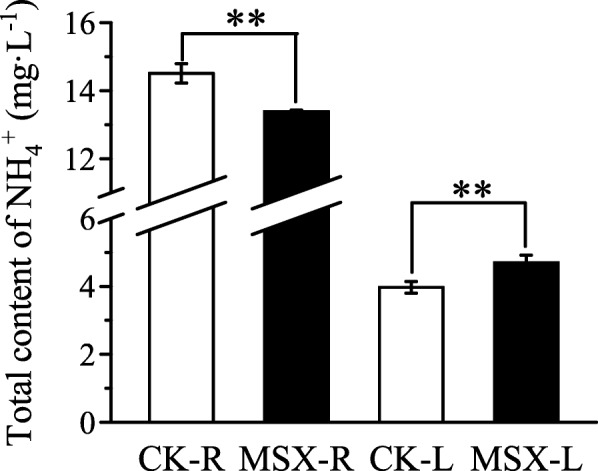
Total content of free ammonium (NH_4_^+^) in control (CK) and methionine sulfoximine (MSX)-treated tea plants. CK-R/CK-L, roots (R) or leaves (L) of tea plants cultured in nutrient solution without MSX; MSX-R/MSX-L, roots (R) or leaves (L) of tea plants subjected to MSX treatment. Asterisks indicate statistically significant differences between MSX and CK groups (**P < 0.01) detected using the Student’s *t*-test

Theanine, also known as L-γ-glutamylethylamide and N5-ethyl-L-glutamine, is a special non-proteinogenic amino acid in tea plant [25, 26]. Interestingly, in this study, the level of theanine enhanced in the leaves after MSX treatment, whereas the major amino acid constituents were all declined (Table 2). To better understand the regulation mechanism, genes related to the theanine metabolism were profiled (Fig. 5). Theanine is mainly synthesized in roots and then transported to leaves. In roots, the biosynthesis of theanine occurs in two steps: glutamine synthesis and ethylamine synthesis. Glutamine synthesis related NAD(P)H-GOGAT encoding genes were mostly inhibited after MSX treatment (Fig. 5). Additionally, two of the three glutamate dehydrogenase (GDH)-encoding unigenes (c128997_g1, c151135_g1, c151135_g2) that were not expressed in leaves, were down-regulated in roots after MSX treatment, except c156729_g1, which showed a 4-fold increment following MSX treatment. The second step of ethylamine synthesis is catalyzed by alanine transaminase (ALT) and arginine decarboxylase (ADC); while the *ALT* gene was inhibited in roots following MSX treatment, *ADC* gene expression showed a contrasting pattern (Fig. 5). Despite its unique brothy or savory (umami) flavor to green tea infusions, theanine is also reportedly involved in the storage or transport of N in a nontoxic form [27]. Products of theanine hydrolysis are utilized in glucose, phenolic, and amino acid metabolism. Ammonium is one of its hydrolyzed products. To reveal the reason why theanine increased in the leaves and considering its whole metabolic process, the total free ammonium content of tea plant was measured. Results showed that the total content of ammonium decreased in roots after MSX treatment but significantly increased in leaves (Fig. 6). We assumed that theanine hydrolysis is controlled by ammonium feedback impact; increase of ammonium resulted from GS inhibition leads to reduced theanine hydrolysis.

### Contents of N-containing and neutral lipids decrease in tea leaves treated with MSX

**Fig. 7:**
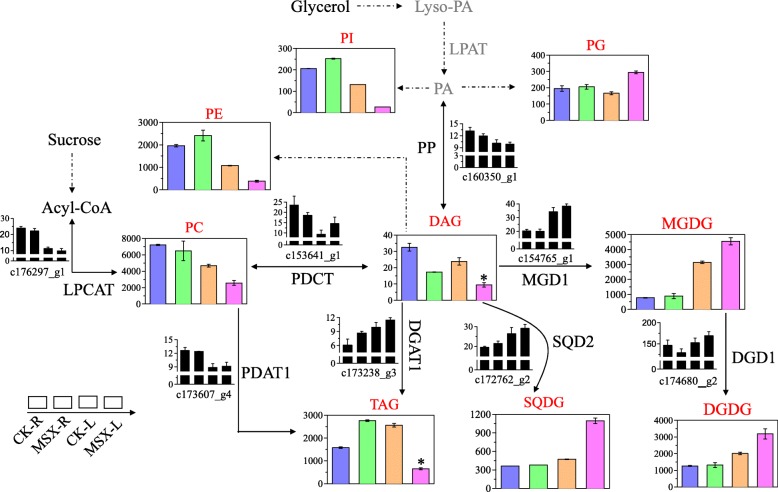
Effect of methionine sulfoximine (MSX) on changes in lipid metabolism. The columns (from left to right) represent samples of control roots (CK-R), MSX-treated roots (MSX-R), control leaves (CK-L) and MSX-treated leaves (MSX-L). Black bars represent the transcription level of genes, and colored bars represent the contents of lipid classes. The content for every lipid class was taken as the sum of species within the given class. Asterisks indicate statistically significant differences between MSX and CK groups (*P < 0.05). PP, phosphatidate phosphatase (EC 3.1.3.4); LPCAT, lysophosphatidylcholine acyltransferase (EC 2.3.1.23); PDCT, diacylglycerol cholinephosphotransferase (EC 2.7.8.2); PDAT1, phospholipid diacylglycerol acyltransferase 1 (EC 2.3.1.158); DGAT1, diacylglycerol O-acyltransferase 1 (EC: 2.3.1.20); MGD1, MGDG synthase 1 (EC 2.4.1.46); DGD1, digalactosyldiacylglycerol synthase 1 (EC 2.4.1.241); SQD2, sulfoquinovosyl transferase (EC 2.4.1.-)

Lipid remodeling had been reported to have correlation with N status and the formation of flavor/aroma origin compounds [11]. Oliveira and Coruzzi [28] have reported that metabolic regulation of GS is associated with the relative abundance of carbon skeleton versus amino acid accumulated in the root tissue. Lipids represent a large part of the carbon skeleton in plants. Therefore, we examined the effect of MSX treatment on lipid content as well as the expression of genes involved in lipid biosynthesis. One hundred twenty-five lipids were identified from this study (Additional file 11: Table S11) and the significantly changed lipids were listed in Table 3 (the percentage of significantly changed lipids are 23.2% for root and 21.6% for leaf). Figure 5 shows the main biosynthetic pathway of lipids. Contents of 2 N-containing phospholipids, phosphatidylcholine (PC), and phosphatidylethanolamine (PE), decreased in MSX-treated leaves compared with control leaves (Fig. 7). Expression of the gene encoding lysophosphatidylcholine acyltransferase (LPCAT; c176297_g1), which is involved in PC biosynthesis, was also decreased in MSX-treated plants compared with the control. Both PC and PE can be synthesized using diacylglycerol (DAG) as a precursor. Our transcriptome data showed that the gene encoding diacylglycerol cholinephosphotransferase (PDCT; c153641_g1) was down-regulated in roots and up-regulated in leaves after MSX treatment; however, the expression of genes involved in PE biosynthesis was not detected following MSX treatment (Fig. 7). Phosphatidylinositol (PI), another member of phospholipids, showed the same tendency as PE.

**Table 3:** Significantly changed lipids in tea roots and leaves treated with MSX compared to that of the control group (CK)

Compounds	Root			Leaf		
	Fold (MSX/CK)	p (t.test)	FDR (q-value)	Fold (MSX/CK)	p (t.test)	FDR (q-value)
34:2 DAG	0.65^a^	0.0006	0.0031	0.38^a^	0.0027	0.01
34:3 DAG	0.76	0.10	0.16	0.33^a^	0.0034	0.0086
36:5 DAG	0.92	0.42	0.42	0.60^a^	0.01	0.02
36:6 DAG	0.73	0.33	0.42	0.60^a^	0.02	0.03
32:1 DGDG	0.81	0.07	1.15	1.67^a^	0.0011	0.02
34:1 MGDG	0.81	0.02	0.10	0.79	0.62	0.62
34:2 MGDG	0.86	0.01	0.17	0.72	0.26	0.67
34:3 MGDG	0.64	0.20	0.52	0.88	0.05	0.32
38:4 MGDG	0.75	0.02	0.13	1.20^a^	0.0039	0.05
36:1 PE	0.98	0.86	0.92	1.29	0.05	0.17
38:1 PE	0.88	0.05	0.35	1.41	0.05	0.22
38:4 PE	1.66	0.04	0.51	2.41	0.01	0.08
40:1 PE	0.86	0.06	0.28	1.36	0.01	0.07
50:3 TAG	0.48^a^	0.0031	0.0096	0.35	0.01	0.06
50:4 TAG	0.60^a^	0.01	0.02	0.54^a^	0.01	0.05
50:5 TAG	0.63^a^	0.0043	0.01	0.55^a^	0.01	0.05
50:6 TAG	0.57^a^	0.02	0.03	0.46^a^	0.0044	0.05
52:3 TAG	2.02	0.54	0.56	0.68^a^	0.0005	0.02
52:5 TAG	0.53^a^	0.0010	0.0061	0.58	0.11	0.19
52:6 TAG	0.39^a^	0.01	0.03	0.37	0.02	0.07
52:7 TAG	0.36^a^	0.0004	0.0047	0.57	0.01	0.06
52:8 TAG	0.21^a^	0.01	0.02	0.99	0.96	0.96
54:4 TAG	3.08	0.46	0.52	0.72	0.03	0.09
54:9 TAG	0.62^a^	0.0014	0.0059	0.45	0.05	0.11
56:5 TAG	0.58^a^	0.0014	0.0053	0.67	0.29	0.43
56:6 TAG	0.62^a^	0.0002	0.0031	0.70	0.42	0.51
56:7 TAG	0.54^a^	0.0013	0.0061	0.67	0.04	0.09
56:8 TAG	0.45^a^	0.0004	0.0040	0.56	0.03	0.08
56:9 TAG	0.58	0.04	0.07	0.49^a^	0.01	0.05
58:2 TAG	2.04	0.49	0.52	0.79^a^	0.01	0.04
58:5 TAG	0.62^a^	0.0042	0.01	0.81	0.24	0.38
58:6 TAG	0.70^a^	0.02	0.03	0.68	0.08	0.15
58:7 TAG	0.43^a^	0.0026	0.0089	0.67	0.02	0.07
60:2 TAG	0.68^a^	0.01	0.02	1.05	0.50	0.58
60:3 TAG	0.67^a^	0.02	0.03	0.85	0.09	0.17
60:4 TAG	0.66^a^	0.0005	0.0036	0.61	0.14	0.23
60:5 TAG	0.70^a^	0.0011	0.0056	0.71	0.07	0.15
60:6 TAG	0.62^a^	0.0001	0.0040	0.36	0.03	0.09
60:7 TAG	0.60^a^	0.0040	0.01	0.43^a^	0.0022	0.04

^a^indicates the significantly changed lipid species

It has been reported that monogalactosyldiaclyglycerol (MGDG), digalactosyl diacylglycerol (DGDG), sulphoquinovosyl diacylglycerol (SQDG) and phosphatidylglycerol (PG) are the four unique lipids that compose the chloroplast membranes [29]. In the present work, contents of these four lipid compounds all increased in leaves when N assimilation was hindered by MSX treatment; however, their levels were unchanged in roots (Fig. 7). The expression of *MGD1* (c154765_g1), *DGD1* (c174680_g2) and *SQD2* (c172762_g2) genes responsible for the biosynthesis of MGDG, DGDG and SQDG, respectively, followed the same trend as the contents of lipids (Fig. 7).

DAG is one of the neutral lipids, and its content decreased in both roots and leaves under MSX treatment (Fig. 7). To synthesize DAG, phosphatidic acid (PA) is catalyzed by phosphatidate phosphatase (PP). The transcription of unigene (c160350_g1) encoding for PP was reduced after MSX treatment (Fig. 7). Triacylglycerol (TAG), another neutral lipid, its content increased in roots of MSX-treated plants and decreased in leaves. TAG is synthesized from either DAG or PC by the action of diacylglycerol O-acyltransferase 1 (DGAT1) or phospholipid diacylglycerol acyltransferase 1 (PDAT1), respectively. Unigene (c173238_g3) encoding for DGAT1 was up-regulated in MSX-treated plants, while PDAT1 encoded unigene (c173607_g4) had no change in the roots and a slight increase in the leaves treated with MSX (Fig. 7).

### Content of catechins decreases in MSX treated tea leaves

Catechins are key metabolites of carbon metabolism in tea plant and determine tea quality [30]. Compared with control leaves, contents of gallocatechin (GC), epigallocatechin (EGC), and catechin (C) isoforms in MSX-treated leaves were significantly reduced, whereas those of gallocatechin gallate (GCG), epigallocatechin gallate (EGCG), epicatechin gallate (ECG), and catechin gallate (CG) were only slightly reduced. By contrast, the content of epicatechin (EC) was slightly increased in MSX-treated leaves compared with control leaves (Table 4). These results suggest that alteration of the N metabolic process through the inhibition of GS activity affects carbon metabolism in tea plant.

**Table 4:** Contents of catechins (mg/g fresh weight) and caffeine in the leaves of tea plant treated with methionine sulfoximine (MSX). The value shown is mean ± standard deviation (SD). CK indicates plants grown without MSX treated, while MSX indicates plants treated with MSX. The asterisk indicates significant difference between MSX and CK group (*P < 0.05, **P < 0.01 in student’s *t*-test). For each treatment, 3 biological replicates were applied

	CK	MSX	P (*t*-test)
Caffeine	0.37 ± 0.03	0.19 ± 0.07*	0.02
Gallocatechin	3.50 ± 0.38	1.02 ± 0.34**	0.0011
Epigallocatechin	12.7 ± 0.94	7.26 ± 2.70*	0.03
Catechin	0.34 ± 0.05	0.19 ± 0.03*	0.0143
Epigallocatechin gallate	26.4 ± 1.59	17.8 ± 6.33	0.09
Epicatechin	0.25 ± 0.01	0.27 ± 0.10	0.83
Gallocatechin gallate	0.17 ± 0.02	0.12 ± 0.04	0.16
Epicatechin gallate	1.37 ± 0.08	0.92 ± 0.33	0.08
Catechin gallate	0.26 ± 0.02	0.19 ± 0.07	0.17

## Discussion

Investigation of short-term inhibition of GS restricting N assimilation without causing N deficiency in tea plant helps to understand the metabolism of tea qualitative compounds. N fertilizer application in tea plantation usually occurs in the soil not the leaf surface; and most of the N is supplied in the form of ammonium (urea hydrolysis) which is assimilated in tea roots [31–34]. Thus, the specific GS inhibitor-MSX was applied through the nutrient solution first to inhibit the assimilation of N in roots and then the resulting affects in tea leaves. This methodology was also adopted in previous studies reported in model plant *Arabidopsis*, where MSX was also applied through the nutrient solution not on the leaf surface to inhibit N assimilation [22]. Application of MSX to tea roots had a stronger effect on root GS than leaf GS (Fig. 1) also indicated that tea roots assimilated most of the N.

Changes in amino acid levels between MSX-treated and control plants were different from those reported in previous long-term N treatment experiments [14, 30], especially for theanine, whose level decreased after the inhibition of GS activity (Table 2). Besides, Liu et al. [35] reported the transcript profiles of genes related to theanine biosynthesis and hydrolysis among different tea plant tissues and cultivars without any treatment, whose findings and insights were totally different from what we do in this study. Both GDH and GS can function in ammonium assimilation [36]. Analysis of *GDH* gene expression (Fig. 5) and free ammonium content (Fig. 6) suggested that GS inhibition enhanced the transcription of *GDH* in tea plant roots which could maintain ammonium assimilation and the biosynthesis of theanine in tea roots, and reduced the reutilization function of GS in tea leaves for the increased accumulation of free ammonium (Fig. 6). This also explained the reason why glutamine level was not directly affected in tea roots but in leaves (Table 1, Fig. 4). Tea plant is an ammonium preferring plant species [14, 15, 31–34]. There is a balance of free ammonium content in higher plants [37, 38]. Both the theanine hydrolysis and the first step of catechins biosynthesis catalyzed by phenylalanine ammonia-lyase in addition to photorespiration can generate ammonium [18, 35]. Ammonium and glutamate are direct products of theanine hydrolysis [35]. The released ammonia is re-assimilated by GS-GOGAT pathway and ammonia might accumulate as a result of GS activity inhibition [37]. Another possible explanation of the increment in ammonium and theanine contents of tea leaves (Fig. 6, Table 2) might be that the hydrolysis of theanine was reduced if hydrolysis is controlled by feedback of products. On the other hand, content of phenylalanine in MSX treated tea leaves also increased (Table 1) while contents of some catechins decreased (Table 4) compared to CK, indicating that generation of ammonium from phenylpropanoid metabolism was also reduced to maintain a relative balance of ammonium as for the reutilization of ammonium by GS was inhibited in tea leaves.

Among the metabolites upstream of amino acid biosynthesis, those related to glycolysis and the TCA cycle were increased after GS repression (Table 1), whereas contents of amino acids such as glycine, serine, isoleucine, threonine, leucine, and valine decreased in the MXS treated group compared with the control group (Fig. 4). We speculate that glycolysis and the TCA cycle affect the biosynthesis of amino acids in a feedback loop. Theanine is the major N-containing molecule in tea [25, 39]. In this study, reduced hydrolysis of theanine (Fig. 5) led to a lower reutilization efficiency of N, resulting in a decline in the contents of other amino acids after GS repression (Fig. 4, Table 2).

Representing a large proportion of N-containing compounds in tea plant, free amino acids not only determine the quality of tea but also are beneficial for human beings. Accumulating evidence showed that the biosynthesis of amino acids is affected by the N status of the tea plant [14, 30]. After the assimilation of N in tea roots, glutamine, theanine, arginine, aspartic acid and glutamate are the most abundant amino acids in the xylem sap [27]. Here we found that inhibition of GS activity by MSX resulted in characteristic changes in the contents of 20 proteinogenic amino acids (Fig. 2). The biosynthesis of serine uses glutamate as a substrate; so its content also decreased in the leaves (Fig. 4). Concentrations of amino acids increased with increasing N supply and abundant N supply promoted the deviation of C flux to amino acids [30]. Hence, the level of amino acid and catechins (represented the C status) were governed by N status. In this study, MSX treatment caused a significant reduction in GS activity both in roots and leaves of tea plants (Fig. 1). The biosynthesis of all 12 free amino acids detected in this study was inhibited, except the non-proteinogenic amino acid theanine, and the biosynthesis of catechins was also inhibited by MSX (Table 2 and Table 4). These results suggest a potential role of GS in the regulation of C and N metabolism at the molecular and metabolic levels. In Arabidopsis, C and amino acids reciprocally modulate the expression of *GS* [28]. Reduction in the contents of amino acids and catechins following the inhibition of GS activity demonstrates a feedback regulation of GS on C and amino acid metabolism.

Most amino acids serve as N sources for secondary metabolism in plants. In tea plant, theanine also functions as N resource incorporating into the downstream pathways like phenolic metabolism after being hydrolyzed by theanine hydrolase [25, 35]. In this study, the content of theanine increased in leaves treated with MSX (Table 1), with a concomitant increase in the level of free ammonium (Fig. 6). Taking into account the processes of theanine biosynthesis, transportation and degradation (Fig. 5), we speculate that GS inhibition reduces the hydrolysis of theanine after its transportation from roots to leaves. This speculation is supported by the reduced biosynthesis of free amino acids in the leaves of tea plants (Fig. 6). Ammonium is not only a product of theanine degradation but also a product of catechin biosynthesis [40]. Catechin, a flavan-3-ol, is produced at the first step of phenylpropanoid metabolism. The content of some catechin isoforms, such as GC, EGC and C, was significantly reduced after MSX treatment, while that of other isoforms was only slightly reduced; however, the content of EC was increased after MSX treatment (Table 4), further explaining the cause of increased content of free ammonium in the leaves. Therefore, GS inhibition leads to a remodeling of tea quality related compounds, including amino acids and catechins in tea plant.

As the major components of biological membranes, lipids serve many functions in plants. MGDG, DGDG, SQDG and PG are the four major glycerolipid components in thylakoid membranes of chloroplast. These lipids are crucial for maintaining the function of chloroplasts not simply because they account for a large fraction of the photosynthetic membranes but because they are assembled into the photosynthetic machinery and are, therefore, directly involved in photosynthetic processes [41]. Here, we found that repression of GS activity increased the contents of MGDG, DGDG, SQDG and PG in leaves, and this increase was concomitant with the increment of gene expression level (Fig. 7.). These results suggest an adaptation of photosynthesis to GS inhibition. Gaude et al. [10] had reported that N deficiency in Arabidopsis affects galactolipid compositions like MGDG, DGDG, with the former decreased but the later increased. However, the treatment condition in our study is different. The inhibition of GS activity within 24 h would not either damage the growth performance of plant or cause N deficiency in plant [21]. In our study, tea plants were treated with the GS inhibitor MSX for a short time (30 min) to inhibit the N metabolism, while avoiding N deficiency [22]. Hence, short-term inhibition of GS in our study and poor N metabolism caused by N deficiency in other studies shared some regulatory network as the changes of DGDG and the related genes both showed an increment in these two treatments. Otherwise, more studies are needed to unravel the regulation network behind the metabolism of photosynthesis lipids under GS inhibition.

A large proportion of the lipids in plants is represented by N-containing phospholipid compounds. In the present work, contents of N-containing lipid compounds PC and PE decreased in leaves upon the inhibition of GS activity (Fig. 7), implying lower N reutilization in tea plant after GS inhibition. It has also been reported that N reutilization in plants has a profound impact on carbohydrate and protein metabolism [42]. Contents of the other lipid compounds, including PI, DAG and TAG, also changed significantly after N availability was hindered by the inhibition of GS activity (Fig. 7). Changes in TAG content in young leaves of tea plant are consistent with a previous study, which reported that N limitation in young leaves leads to low accumulation of TAG [11]. In this study, levels of phospholipids, glycolipids, and neutral lipids changed in leaves after GS inhibition, suggesting that alteration of N status in plants affects lipid speciation [42]. Our findings are consistent with the view that plants remodel C-N balance to maintain their growth performance as well as metabolism [43].

## Conclusions

In this study, we determined the effect of GS activity on changes in the transcriptome of tea plants. Combined with the metabolic profile analysis, the contribution of GS activity to the biosynthesis of two main flavor and aroma origin compounds of tea plant, namely free amino acids and lipids, was further unraveled. The inhibition of GS in tea plant resulted in the reprogramming of metabolic pathways, which would further affect the formation of tea quality. Therefore, our results provided novel insights into the metabolic pathways of tea plant, an ammonium preferring plant species, in response to repressed GS activity.

## Methods

### Plant material and treatment

Rooted-cuttings of the tea cultivar ‘Longjing 43’, a nationally released and commercially available clone, were collected from a tea plantation owned by the Tea Research Institute, Chinese Academy of Agricultural Sciences; no permission was needed for the collection of these materials. One-year-old rooted cuttings of ‘Longjing 43’ were first pre-cultured in a dilute nutrient solution for 1 month to maintain the growth of young tea plants, especially the roots, as described previously [15]. Subsequently, tea cuttings were transferred to pots (8 plants per pot) containing 4.5 L of full-strength nutrient solution for 1 week before the experimental treatment started. One week later, plants were transferred to the same fresh nutrient solution without or with 0.1 mmol/L MSX (sigma 5379) to repress the assimilation of N in tea plant for 30 min [20, 22, 23, 44, 45]; tea plants not treated with MSX served as a control (CK). Three pots were used for each treatment, and each pot was considered a biological replicate. To minimize the deviations caused by individual tea plants, newly expanded young leaves and absorbing roots from eight plants of the same pot were collected and mixed together as a sample pool for one biological replicate, respectively. Roots and leaves were sampled after 30 min of MSX or control treatment, quickly frozen in liquid nitrogen, and stored at − 80 °C for physiological, transcriptome and metabolite analyses.

The full-strength nutrient solution used in this study contained the following macronutrients (mmol/L): (NH_4_)_2_SO_4_ (1.0), Ca (NO_3_)_2_ (0.5), KH_2_PO_4_ (0.1), K_2_SO_4_ (0.5), MgSO_4_ (0.4), CaCl_2_ (0.3) and micronutrients (μmol/L): H_3_BO_3_ (10), MnSO_4_ (1.5), ZnSO_4_ (1.0), CuSO_4_ (0.2), (NH_4_)_6_Mo_7_O_24_ (0.07) and Fe-EDTA (6.3). The pH of all the nutrient solutions was continuously titrated to 5.0 with H_2_SO_4_ and NaOH. All experiments were carried out in an environmentally controlled growth chamber under the following conditions: 26 °C/22 °C day/night temperature, 70% relative humidity, 14 h light/10 h dark photoperiod, and 200 μmol m^− 2^ s^− 1^ light intensity.

### Determination of GS activity

To determine the total GS activity, 100 mg of each frozen sample was ground to fine powder using a ball miller (M301 Retsch, Germany). The measurement was performed according to Magalhaes and Huber [46] with minor modifications. Briefly, samples were homogenized in extraction buffer (50 mmol/L Tris-HCl [pH 8.0], 2 mmol/L MgSO_4_, 4 mmol/L dithiothreitol, and 0.4 mmol/L sucrose). Plant extracts were centrifuged at 13000 g (4 °C) for 25 min and the supernatant of each extract was analyzed to determine the soluble protein content [47] and GS activity. GS activity was determined after incubating the supernatants in a reaction buffer (100 mmol/L Tris-HCl, 80 mmol/L MgSO_4_, 20 mmol/L sodium glutamate, 80 mM NH_2_OH, 20 mmol/L cysteine, 2 mmol/L EGTA and 40 mmol/L ATP) at 37 °C for 30 min [46, 48]. Then, a stop solution containing 0.2 mol/L TCA, 0.37 mol/L FeCl_3_ and 0.6 mol/L HCl was added; and the absorbance of supernatants at 540 nm was recorded in a spectrophotometer. Specific activity of GS was expressed as the absorbance (A) of mg^− 1^ protein·h^− 1^.

### RNA preparation, library construction, transcriptome assembly and data processing

Total RNA was isolated from all biological replicates (three biological replicates for each treatment) using Trizol reagent (Invitrogen, USA). After RNA quantification and qualification, a total amount of 1.5 μg RNA per sample was used for cDNA library construction following the manual of NEBNext® Ultra™ RNA Library Prep Kit for Illumina® (NEB, USA). The sequencing was performed on an Illumina Hiseq platform (Novogene Bioinformatics Technology Co. Ltd., Beijing) and paired-end reads were generated.

High quality reads (clean reads) were obtained by removing low-quality reads with ambiguous nucleotides, and adaptor sequences were filtered from the raw reads. Transcriptome assembly was accomplished based on the left.fq and right.fq using Trinity with min_kmer_cov set to 2 and all other parameters set default [49]. Functional annotation of the obtained unigenes was conducted using the databases of NCBI non-redundant nucleotide sequences (Nt), NCBI non-redundant protein sequences (Nr), Clusters of Orthologous Groups of proteins (KOG/COG), Protein family (Pfam), a manually annotated and reviewed protein sequence database (Swiss-Prot), Gene Ontology (GO), and KEGG Ortholog (KO) [50]. Otherwise, all the acquired unigenes were also annotated with latest comprehensively annotated tea plant genome (*Camellia sinensis var. sinensis*) (Tea plant CSS improved gene annotation Version2, Release Date: 2019-01-18) [51, 52].

For each sample, gene expression levels were calculated by RSEM [53]. DESeq R package (1.10.1) was applied to dissect the differentially expressed genes (DEGs) of treated (MSX) vs. control (CK) groups. DESeq provides statistical routines for determining differential expression in digital gene expression data using a model based on the negative binomial distribution. The resulting *P* values were adjusted using the Benjamini and Hochberg’s approach for controlling the false discovery rate (FDR). In this study, three biological replicates were applied, so genes with an adjusted *P*-value (P adj) < 0.05 found by DESeq were assigned as differentially expressed. P adj is an adjusted *p*-value, taking in to account the FDR. Applying a FDR becomes necessary when we’re measuring thousands of variables (e.g. gene expression levels) from a small sample set (e.g. a couple of individuals). A p-value of 0.05 implies that we are willing to accept that 5% of all tests will be false positives. An FDR-adjusted p-value (aka a q-value) of 0.05 implies that we are willing to accept that 5% of the tests found to be statistically significant (e.g. by p-value) will be false positives. Such an adjustment is necessary when we’re making multiple tests on the same sample. The GOseq R packages based Wallenius non-central hyper-geometric distribution was used for the GO enrichment analysis of the DEGs [54]. The biological interpretation of the DEGs was further completed by assigning them to metabolic pathways using the Kyoto Encyclopaedia of Genes and Genomes (KEGG) annotation. All the transcriptome data had been submitted to the NCBI Sequence Read Archive (SRA) database under the accession number of SRR5992801 (Released date 31/08/2018).

### Metabolomics and lipidomics analysis with GC/LC-MS

The metabolites were extracted according to Giavalisco et al. [55]. Fresh samples were first lyophilized and then send to Max Planck Institute of Molecular Plant Physiology (MPIMP) where the measurement was done. Briefly, 50 mg for leaf sample (25 mg for root sample) was homogenized in 1 ml MTBE buffer, with incubating for 10 min at 4 °C on an orbital shaker, followed by another 10 min incubation in an ultra-sonication bath. After adding 0.5 ml of UPLC-grade methanol: water 1: 3, the homogenate was vortexed and spun for 5 min at 4 °C in a table-top centrifuge (Eppendorf). The addition of methanol: water led to a phase separation, providing the upper organic phase, containing the lipids, a lower aqueous phase, containing the polar and semipolar metabolite.

The samples for primary metabolites were measured with an Agilent Technologies Gas Chromatography (GC) coupled to a Leco Pegasus HT mass spectrometer which consists of an Electron Impact ionization source (EI) and a Time of Flight (TOF) mass analyzer. After extraction from the chromatograms, the data was processed, aligned, filtered and annotated as Giavalisco et al. [55] described. The significance of each metabolite was evaluated by the Student’s *t*-test (*P* < 0.05) and FDR (q-value < 0.05). FDRs were calculated in a hierarchical way according to the metabolite groups.

Samples for lipids were measured using the Waters ACQUITY Reversed Phase Ultra Performance Liquid Chromatography (RP-UPLC) coupled to a Thermo-Fisher Exactive mass spectrometer which consists of an ElectroSpray Ionization source (ESI) and an Orbitrap mass analyzer. Peak annotation and data processing was accomplished according to Liu et al. [11]. After combining the lipid compounds detected in positive and negative electrospray ionization modes, R software was applied for data normalization. Firstly, we calculated the coefficient of variation from raw chromatogram intensities for each lipid compound. Secondly, the intensities of the compounds with 50% lower variation, excluding TAGs, were used to normalize all the lipids in the data set. The sum of species within the given class was calculated based on the content of every class. Statistical analysis using student’s *t*-test (*P* < 0.05) was performed to identify significantly changed lipid classes between MSX and CK groups. Student’s t-test and FDR were applied to identify significantly changed lipid species between MSX and CK groups.

### Measurement of catechins and free amino acids

Catechins in samples were extracted with 75% methanol and 0.1% formic acid in an ultrasonic bath for 15 min at room temperature. The extracts were then filtered through a 0.22 μm PTFE filter before measurement. Quantification of catechins was performed on a reverse phase high-performance liquid chromatographic (HPLC) system using a C18 reverse-phase column (250 × 4.6 mm i.d., Phenomenex, Torrance, CA,USA) as described previously [56]. Free amino acids were analyzed by HPLC with an AccQ Tag column (3.9 × 150 mm) and fluorescence detection was performed after derivatization with AccQFluor Reagent Kit (Waters Corporation) following the manufacturer’s manual [14].

### Tissue ammonium measurement

Tissue ammonium was measured as described by Weatherburn [57]. 0.5 g of each sample was homogenized in 5 ml sulfuric acid solution (0.3 mM) to extract the ammonium of tea samples. After centrifuge the homogenate at 20000 g (4 °C) for 20 min, 0.2 ml of the supernatant was transferred to a 10 ml centrifuge tube, followed by addition of 4.9 ml phenol solution (containing 0.5% w/w sodium nitroprusside) and 4.9 ml alkaline sodium hypochlorite solution (5 g NaOH dissolved in 52.5 g/L sodium hypochlorite solution), and mixed. Absorbance of the mixture at 625 nm was recorded in a spectrophotometer. Ammonium content was calculated based on the standard curve, which was generated using ammonium sulfate solution.

Student’s *t*-test (*P* < 0.05) was performed for the statistical analysis of GS activity and ammonium determination.

## Supplementary information

**Additional file 1: Table S1.** Summary of transcriptome data obtained from tea plants treated with or without methionine (MSX) using Illumina HiSeq.

**Additional file 2: Table S2.** Results of de novo assembly of transcriptome data and tea plant unigenes.

**Additional file 3: Table S3.** The differentially expressed genes (DEGs) in roots of methionine sulfoximine (MSX) treated tea plants compared with roots of untreated (control) plants.

**Additional file 4: Table S4.** The differentially expressed genes (DEGs) in leaves of methionine sulfoximine (MSX) treated and untreated (control) plants.

**Additional file 5: Table S5.** GO enrichment analysis of the differentially expressed genes in MSX treated tea roots vs. non treated tea roots.

**Additional file 6:Table S6.** GO enrichment analysis of the differentially expressed genes in MSX treated tea leaves vs. non treated tea leaves.

**Additional file 7:Table S7.** KEGG enrichment of the differentially expressed genes in tea roots of MSX vs. CK groups.

**Additional file 8:** **Table S8.** KEGG enrichment of the differentially expressed genes in tea leaves of MSX vs. CK groups.

**Additional file 9:** **Table S9.** KEGG enrichment analysis of more than five differentially expressed genes (DEGs) in roots of methionine sulfoximine (MSX) treated tea plants compared with roots of untreated (control) plants.

**Additional file 10:** **Table S10.** List of 155 primary metabolites identified in this study.

**Additional file 11:Table S11.** List of 125 lipid compounds identified in this study and the fold-change in each lipid between methionine sulfoximine treated (MSX) and control (CK) groups.

## Data Availability

All data generated or analysed during this study are included in this published article and its supplementary information files.

## Abbreviations

Ala: Alanine
Asp: Asparate
C: Catechin
CG: Catechin gallate
Cys: Cysteine
DAG: Diacylglycerol
DEGs: Differentially expressed genes
DGAT1: Diacylglycerol O-acyltransferase 1
DGDG: Digalactosyl diacylglycerol
EC: Epicatechin
ECG: Epicatechin gallate
EGC: Epigallocatechin
EGCG: Epigallocatechin gallate
GC: Gallocatechin
GC: Gas chromatography
GCG: Gallocatechin gallate
Glu: Glutamic acid
GO: Gene ontology
GOGAT: Glutamine-2-oxoglutarate amidotransferase
GS: Glutamine synthetase
KEGG: Kyoto encyclopaedia of genes and genomes
Leu: Leucine
Lys: Lysine
Met: Methionine
MGDG: Monogalactosyldiaclyglycerol
MSX: Methionine sulfoximine
N: Nitrogen
PA: Phosphatidic acid
PC: Phosphatidylcholine
PDAT1: Phospholipid diacylglycerol acyltransferase 1
PDCT: Diacylglycerol cholinephosphotransferase
PE: Phosphatidylethanolamine
PG: Phosphatidylglycerol
Phe: Phenylalanine
PI: Phosphatidylinositol
PP: Phosphatidate phosphatase
Ser: Serine
SQDG: Sulphoquinovosyl diacylglycerol
TAG: Triacylglycerol
Thea: Theanine
Thr: Threonine
Tyr: Tyrosine
Val: Valine
