# Supplementary material for: Short-term inhibition of glutamine synthetase leads to reprogramming of amino acid and lipid metabolism in roots and leaves of tea plant (Camellia sinensis L.)
Source: BMC Plant Biol. 2019 Oct 15;19:425. doi: 10.1186/s12870-019-2027-0 (PMC6794879; doi:10.1186/s12870-019-2027-0)
Supplement: Supplementary file 1 — Additional file 1: Table S1. Summary of transcriptome data obtained from tea plants treated with or without methionine (MSX) using Illumina HiSeq. [file 12870_2019_2027_MOESM1_ESM.docx]

Table S1. Summary of transcriptome data obtained from tea plants treated with or without methionine (MSX) using Illumina HiSeq.

| Sample | No. of raw reads | No. of clean reads | No. of clean bases |
| --- | --- | --- | --- |
| CK_R1^a^ | 46,381,204 | 45,147,804 | 6.77G |
| CK_R2 | 56,421,588 | 54,953,988 | 8.24G |
| CK_R3 | 49,161,472 | 47,920,246 | 7.19G |
| CK_L1^b^ | 53,854,980 | 52,522,944 | 7.88G |
| CK_L2 | 53,273,474 | 52,001,930 | 7.8G |
| CK_L3 | 59,206,118 | 57,778,630 | 8.67G |
| MSX_R1^c^ | 50,134,298 | 48,917,650 | 7.34G |
| MSX_R2 | 52,002,794 | 50,580,428 | 7.59G |
| MSX_R3 | 58,963,916 | 57,378,106 | 8.61G |
| MSX_L1^d^ | 65,347,786 | 63,849,132 | 9.58G |
| MSX_L2 | 60,156,036 | 58,579,014 | 8.79G |
| MSX_L3 | 58,703,292 | 57,324,898 | 8.6G |

^a^ Roots and ^b^ leaves form tea plants grown under control condition, ^c^ Roots and ^d^ leaves form tea plants treated with methionine sulphoximine.
