# Supplementary material for: Short-term inhibition of glutamine synthetase leads to reprogramming of amino acid and lipid metabolism in roots and leaves of tea plant (Camellia sinensis L.)
Source: BMC Plant Biol. 2019 Oct 15;19:425. doi: 10.1186/s12870-019-2027-0 (PMC6794879; doi:10.1186/s12870-019-2027-0)
Supplement: Supplementary file 2 — Additional file 2: Table S2. Results of de novo assembly of transcriptome data and tea plant unigenes. [file 12870_2019_2027_MOESM2_ESM.docx]

Table S2. Results of *de novo* assembly of transcriptome data and tea plant unigenes.

| Unigene length interval | 200-500 bp | 500-1 kbp | 1 k-2 kbp | >2 kbp | Total |
| --- | --- | --- | --- | --- | --- |
| Number of Unigenes | 231242 | 58016 | 25249 | 11662 | 326169 |
