# Supplementary material for: Short-term inhibition of glutamine synthetase leads to reprogramming of amino acid and lipid metabolism in roots and leaves of tea plant (Camellia sinensis L.)
Source: BMC Plant Biol. 2019 Oct 15;19:425. doi: 10.1186/s12870-019-2027-0 (PMC6794879; doi:10.1186/s12870-019-2027-0)
Supplement: Supplementary file 9 — Additional file 9: Table S9. KEGG enrichment analysis of more than five differentially expressed genes (DEGs) in roots of methionine sulfoximine (MSX) treated tea plants compared with roots of untreated (control) plants. [file 12870_2019_2027_MOESM9_ESM.docx]

**Table S9.** KEGG enrichment analysis of more than five differentially expressed genes (DEGs) in roots of methionine sulfoximine (MSX) treated tea plants compared with roots of untreated (control) plants.

| KEGG pathway (Class) | KEGG pathway (term) | ID | Gene number |
| --- | --- | --- | --- |
| Genetic Information Processing  (Translation) | Ribosome | ko03010 | 383 |
|  | Ribosome biogenesis in eukaryotes | ko03008 | 22 |
|  | RNA transport | ko03013 | 44 |
|  | Aminoacyl-tRNA biosynthesis | ko00970 | 9 |
| Genetic Information Processing  (Transcription) | Spliceosome | ko03040 | 30 |
| Genetic Information Processing  (Folding, sorting and degradation) | Proteasome | ko03050 | 18 |
|  | Protein processing in endoplasmic reticulum | ko04141 | 94 |
|  | RNA degradation | ko03018 | 18 |
|  | Protein export | ko03060 | 11 |
| Cellular Processes  (Transport and catabolism) | Lysosome | ko04142 | 62 |
| Cellular Processes  (Cell growth and death) | Cell cycle | ko04110 | 26 |
| Energy metabolism | Carbon fixation in photosynthetic organisms | ko00710 | 27 |
|  | Methane metabolism | ko00680 | 11 |
|  | Sulfur metabolism | ko00920 | 5 |
|  | Oxidative phosphorylation | ko00190 | 64 |
|  | Nitrogen metabolism | ko00910 | 16 |
| Global and overview maps | Carbon metabolism | ko01200 | 88 |
|  | Biosynthesis of amino acids | ko01230 | 60 |
|  | 2-Oxocarboxylic acid metabolism | ko01210 | 21 |
|  | Fatty acid metabolism | ko01212 | 20 |
| Carbohydrate metabolism | Glycolysis / Gluconeogenesis | ko00010 | 37 |
|  | Glyoxylate and dicarboxylate metabolism | ko00630 | 31 |
|  | Citrate cycle (TCA cycle) | ko00020 | 30 |
|  | Pyruvate metabolism | ko00620 | 24 |
|  | Starch and sucrose metabolism | ko00500 | 16 |
|  | Pentose phosphate pathway | ko00030 | 14 |
|  | Propanoate metabolism | ko00640 | 14 |
|  | Amino sugar and nucleotide sugar metabolism | ko00520 | 12 |
|  | Pentose and glucuronate interconversions | ko00040 | 10 |
|  | Fructose and mannose metabolism | ko00051 | 10 |
|  | Ascorbate and aldarate metabolism | ko00053 | 9 |
| Amino acid metabolism | Arginine and proline metabolism | ko00330 | 30 |
|  | Valine, leucine and isoleucine degradation | ko00280 | 26 |
|  | Cysteine and methionine metabolism | ko00270 | 26 |
|  | Glutathione metabolism | ko00480 | 25 |
|  | Alanine, aspartate and glutamate metabolism | ko00250 | 20 |
|  | Phenylalanine metabolism | ko00360 | 18 |
|  | beta-Alanine metabolism | ko00410 | 12 |
|  | Lysine degradation | ko00310 | 12 |
|  | Tyrosine metabolism | ko00350 | 11 |
|  | Tryptophan metabolism | ko00380 | 11 |
|  | Cyanoamino acid metabolism | ko00460 | 7 |
|  | Phenylalanine, tyrosine and tryptophan biosynthesis | ko00400 | 7 |
|  | Histidine metabolism | ko00340 | 6 |
|  | Glycine, serine and threonine metabolism | ko00260 | 5 |
| Lipid metabolism | Fatty acid degradation | ko00071 | 21 |
|  | Sphingolipid metabolism | ko00600 | 13 |
|  | Glycerolipid metabolism | ko00561 | 11 |
|  | alpha-Linolenic acid metabolism | ko00592 | 10 |
|  | Glycerophospholipid metabolism | ko00564 | 9 |
|  | Arachidonic acid metabolism | ko00590 | 8 |
|  | Biosynthesis of unsaturated fatty acids | ko01040 | 8 |
|  | Steroid biosynthesis | ko00100 | 7 |
|  | Fatty acid biosynthesis | ko00061 | 6 |
|  | Ether lipid metabolism | ko00565 | 5 |
| Biosynthesis of other secondary metabolites | Phenylpropanoid biosynthesis | ko00940 | 17 |
|  | Isoquinoline alkaloid biosynthesis | ko00950 | 5 |
| Nucleotide metabolism | Purine metabolism | ko00230 | 19 |
| Environmental Information Processing  (Signal transduction) | Calcium signaling pathway | ko04020 | 28 |
|  | Plant hormone signal transduction | ko04075 | 5 |
| Environmental adaptation | Plant-pathogen interaction | ko04626 | 23 |
| Environmental Information Processing; Membrane transport | ABC transporters | ko02010 | 5 |
